# Identification of epigenetic factor KAT2B gene variants for possible roles in congenital heart diseases

**DOI:** 10.1042/BSR20191779

**Published:** 2020-04-15

**Authors:** Yong-Sheng Hou, Jing-Zhi Wang, Shuai Shi, Ying Han, Yue Zhang, Ji-Xin Zhi, Chao Xu, Fei-Feng Li, Gui-Yu Wang, Shu-Lin Liu

**Affiliations:** 1 Genomics Research Center (Part of the State-Province Key Laboratory of Biopharmaceutical Engineering, China), College of Pharmacy, Harbin Medical University, Harbin, China; 2 The First Affiliated Hospital of Harbin Medical University, Harbin, China; 3 Department of Cardiology, The Fourth Affiliated Hospital of Harbin Medical University, Harbin, China; 4 Translational Medicine Research and Cooperation Center of Northern China, Heilongjiang Academy of Medical Sciences, Harbin, China; 5 Department of Colorectal Surgery of The Second Affiliated Hospital, Harbin Medical University, Harbin, China; 6 Department of Microbiology, Immunology and Infectious Diseases, University of Calgary, Calgary, Canada

**Keywords:** Congenital heart disease, epigenetic factors, Hardy-Weinberg equilibrium test, KAT2B

## Abstract

Congenital heart disease (CHD) is a group of anatomic malformations in the heart with high morbidity and mortality. The mammalian heart is a complex organ, the formation and development of which are strictly regulated and controlled by gene regulatory networks of many signaling pathways such as TGF-β. KAT2B is an important histone acetyltransferase epigenetic factor in the TGF-β signaling pathway, and alteration in the gene is associated with the etiology of cardiovascular diseases. The aim of this work was to validate whether *KAT2B* variations might be associated with CHD. We sequenced the *KAT2B* gene for 400 Chinese Han CHD patients and evaluated SNPs rs3021408 and rs17006625. The statistical analyses and Hardy–Weinberg equilibrium tests of the CHD and control populations were conducted by the software SPSS (version 19.0) and PLINK. The experiment-wide significance threshold matrix of LD correlation for the markers and haplotype diagram of LD structure were calculated using the online software SNPSpD and Haploview software. We analyzed the heterozygous variants within the CDS region of the *KAT2B* genes and found that rs3021408 and rs17006625 were associated with the risk of CHD.

## Introduction

Congenital heart disease (CHD) is a group of anatomic malformations in the heart and is the most common type of birth defects with high morbidity and mortality [1]. There are two main categories of CHD, cyanotic and acyanotic, with the former including tetralogy of Fallot (TOF), truncus arteriosus (TA) and transposition of the great arteries (TGA), and the latter including ventricular septal defect (VSD), atrioventricular septal defect (AVSD) and patent ductus arteriosus (PDA) [2,3]. Many CHDs are complicated by arrhythmias or heart failure or both [4]. The worldwide prevalence of CHD ranges from 1 to 150 per 1000 neonates [5,6], about 1% of which requires clinical intervention [7].

CHDs are multifactorial heart diseases, involving many genetic variations in development, mostly not of the Mendel’s inheritance mode [8,9], including chromosomal variants such as the 22q11 deletion [6,10]. Over the past decades, numerous genetic defects have been reported for their associations with sporadic or familial CHD cases, leading to better understanding of CHDs [11,12], but the genetic abnormalities of etiological significance for most CHDs remain largely unknown.

In a previous study, we demonstrated the associations of the variant rs2289263 upstream of the *SMAD3* gene 5’UTR with increased risk of VSD and variants in the *Lefty* gene (rs2295418 in *Lefty2* and rs360057 in *Lefty1*) with the risk of other CHD types [2]. LEFTY and SMAD3 both play central roles in the TGF-β signaling pathway [13,14], which is involved in the development of the mammalian heart during embryogenesis together with numerous transcription and epigenetic factors [2,15,16].

KAT2B is an important histone acetyltransferase (HAT) epigenetic factor in the TGF-β signaling pathway. Alteration in epigenetic regulation of gene expression in vascular cells has been associated with the etiology of cardiovascular diseases [17,18]. KAT2B can acetylate the exposed lysines for the modification of histones [19,20]. It is also an important nuclear epigenetic factor that binds to many sequence-specific factors involved in cell growth or differentiation [19]. *KAT2B* gene variations may lead to vascular disorders and coronary heart abnormalities [21,22].

Based on the documented key roles of KAT2B in the TGF-β signaling pathway and vascular cellular processes, we postulate its associations with CHDs. In the present study, we analyzed the transcribed region and splicing sites of the *KAT2B* gene and compared the gene sequences between 400 Chinese Han CHD patients and 420 controls. We found that rs3021408 and rs17006625 in the CDS (coding sequence) region of *KAT2B* gene were associated with the risk of CHDs.

## Materials and methods

### The study population

From the First, Second and Fourth Affiliated Hospitals of Harbin Medical University, Harbin, China, we collected specimens of 400 CHDs patients, and from the Medical Examination Center of the Second Affiliated Hospital of Harbin Medical University, 420 normal controls with no reported cardiac phenotypes or defects were recruited (Table 1). All the CHD patients and normal controls received comprehensive physical examination, electrocardiogram and ultrasonic echocardiogram examinations. None of the patients showed any other cardiac or systematic abnormalities, and the normal controls did not show any defects in the heart or other body parts. Before this work, we had obtained a written informed consent from each participant or their parents on behalf of minors, and the Ethics Committee of the Harbin Medical University approved this work, consistent with the 1975 Declaration of Helsinki.

**Table 1:** Clinical characteristics and analysis of the study population

Parameter	CHD	Control	*P*
**Sample (*n*)**	400	420	–
**Male/Female (*n*)**	179/221	183/237	
**Age (years)**	15.53 ± 18.10	14.98 ± 9.86	0.207

Data are shown as mean ± SD between the two groups; there were no statistical differences of the age and gender composition.

### DNA and SNP genotyping analysis

As detailed in previous studies [23,24], the genomic DNA was extracted from the peripheral blood leukocytes of the participants. The human *KAT2B* gene consisting of 17 exons is located on 3p24.3. In order to determine the genotypes of the *KAT2B* gene, we amplified the 17 exons and the splicing sites of the gene using polymerase chain reaction (PCR) method (Supplementary Table S1). All reagents were purchased from the TransGen Biotech company, Beijing, China. The PCR reaction system was 10 × PCR buffer: 5.0 μl, dNTP mixture (2.5 μmol/l): 1.0 μl, rTaq dnase 0.4 μl, up/down primers (10 μmol/l each): 1.0 μl, DNA: 150 ng, add purified water to 50 μl. The products were sequenced using standard protocols [2,25]. Then, the genotypes were determined using PCR (Supplementary Table S2) and gene sequencing methods [26,27].

### Statistical analysis

We determined genotypes rs3021408, rs17006625, rs148960024 and rs41285059 within the CDS region of the *KAT2B* gene (Supplementary Figure S1A) on 400 CHDs patients and 420 normal controls. Using the software SPSS (version 19.0) and PLINK, we carried out the statistical analyses and Hardy–Weinberg equilibrium tests of the CHDs and control populations as previously reported [23,27]. The experiment-wide significance threshold, matrix of linkage-disequilibrium (LD) correlation for the markers and haplotype diagram of LD structure were calculated using the Haploview software as previously reported [27].

### Multiple sequence alignments

The KAT2B protein sequences of various species were obtained from the NCBI website (http://www.ncbi.nlm.nih.gov/), and multiple-sequence alignments and position of the SNPs in the KAT2B protein were carried out using the Vector NTI software [28].

## Results

### Patients

We recruited the participants from the First, Second and Fourth Affiliated Hospitals of Harbin Medical University and confirmed the clinical diagnosis for each of the patients. They had no history or manifestations of any other disease or systemic abnormalities except CHDs. We also established that their mothers did not take medications or attract infections during her gestation, because such factors have been reported to be associated with heart malformation in pregnancy [29,30].

The 400 CHD patients included 184 with ventricular septal defects (VSD), 131 with atrial septal defects (ASD), 54 with patent ductus arteriosus (PDA), 7 with tetralogy of Fallot, 4 with pulmonary stenosis, and 19 with other types of congenital heart defects. The 400 CHD patients (male 179, female 221, with an average age of 15.42 years) and 420 unrelated controls (male 183, female 237, with an average age of 14.02 years) recruited for the present study had no statistical differences in gender composition or age between the two groups (Table 1).

### Genotype analysis of the *KAT2B* gene

We sequenced the *KAT2B* gene to determine whether genetic variants in the *KAT2B* gene may confer susceptibility to CHDs. In comparisons of the transcribed regions and splicing sites of *KAT2B* between the patients and controls, we identified variations rs3021408, rs17006625, rs148960024 and rs41285059 within the CDS region of the *KAT2B* gene (Supplementary Figure S1A). Further analysis showed that the genetic heterozygosity was very low in rs148960024 and rs41285059, but remarkably high in rs3021408 and rs17006625 (Supplementary Figure S1B).

The genotype and allele distributions of rs3021408 and rs17006625 in the CHD patients and control subjects were shown in Supplementary Table S3. Chi-Square tests showed that the variants rs3021408 and rs17006625 were associated with the risk of CHDs in the Chinese Han population (Table 2). We conducted the Hardy–Weinberg equilibrium test for the CHDs and controls and it was in line with the equilibrium (Table 3).

**Table 2:** SNP rs3021408 and rs17006625 in the CDS region of *KAT2B* gene were associated with the risk of congenital heart diseases in Chinese populations

Comparison SNP	Type	Pearson Chi-square				Spearman Correlation			
		Value	Min count^1^	df	Asymp. Sig. (2-sided)	Value	Asymp. Std. error^2^	Approx. T^3^	Approx. Sig
rs3021408	**Genotype**	11.667^1^	87.8	2	**0.003**	0.117	0.035	3.373	**0.001** ^4^
	**Allele**	11.513^1^	369.76	1	**0.004**	0.084	0.025	3.403	**0.001** ^4^
rs17006625	**Genotype**	5.682^1^	5.37	2	0.058	0.038	0.034	2.379	**0.018** ^4^
	**Allele**	5.461^1^	103.90	1	**0.019**	0.058	0.024	2.339	**0.019** ^4^

1 The minimum expected count.

2 Not assuming the null hypothesis.

3 Using the asymptotic standard error assuming the null hypothesis.

4 Based on normal approximation.

**Table 3:** Hardy–Weinberg equilibrium test for the study population groups

SNPs	Genotype	H–W equilibrium testing		
	Homo/Hetero/Homozygote	O(HET)	E (HET)	P
**rs3021408**	180/398/242	0.4854	0.4971	0.5272
**rs17006625**	11/191/618	0.2329	0.226	0.4424

**Figure 1:**
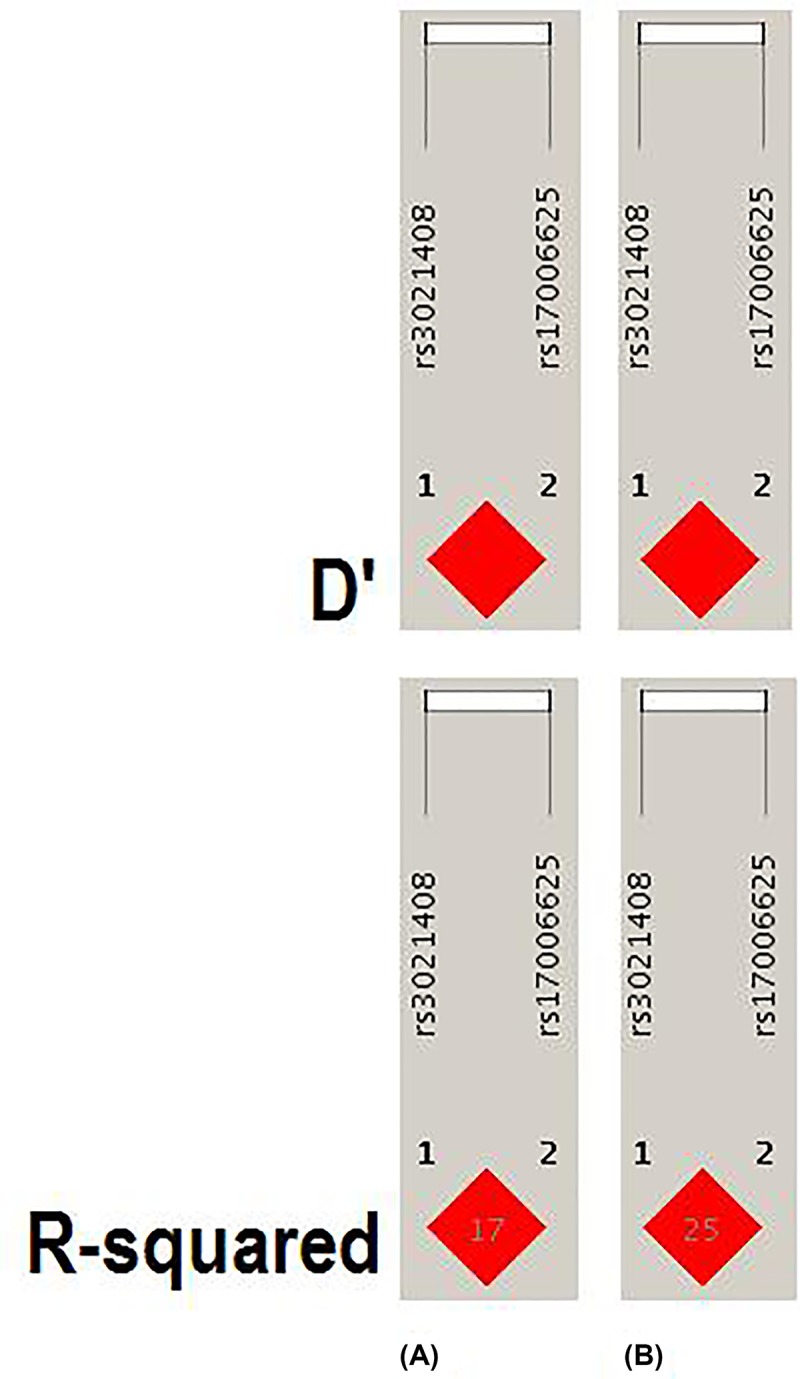
LD analysis of the variants rs3021408 and rs17006625 in the *KAR2B* gene (**A**) Data analysis of CHD patients and controls from the present study of variants in *KAR2B* gene. (**B**) Data from HapMap CHB of variants in *KAR2B* gene. The data from the HapMap CHB and this work were consistent.

The experiment-wide significance threshold of the variants rs3021408 and rs17006625 in *KAT2B* gene was 0.027. The Haploview software was used to conduct LD analysis of the variants rs3021408 and rs17006625, and the results were consistent with the data from the HapMap CHB population (Figure 1). The genotype frequencies in the CHD and control groups were further analyzed by three genetic models, including trend, dominant and recessive models, in addition to Chi-square and Fisher tests (Table 4). All those analyses indicated that the variants rs3021408 and rs17006625 were associated with the risk of CHDs.

**Table 4:** SNP rs3021408 and rs17006625 *within KAT2B* gene associated with the risk of congenital heart diseases

SNPs	Trend model	Dominant model	Recessive model
**rs3021408**	0.0007977^1^	0.01439^1^	0.002358^1^
**rs17006625**	0.01762^1^	0.01892^1^	0.5474

1 statistically significant.

### Conservation of the protein in evolution

**Figure 2:**
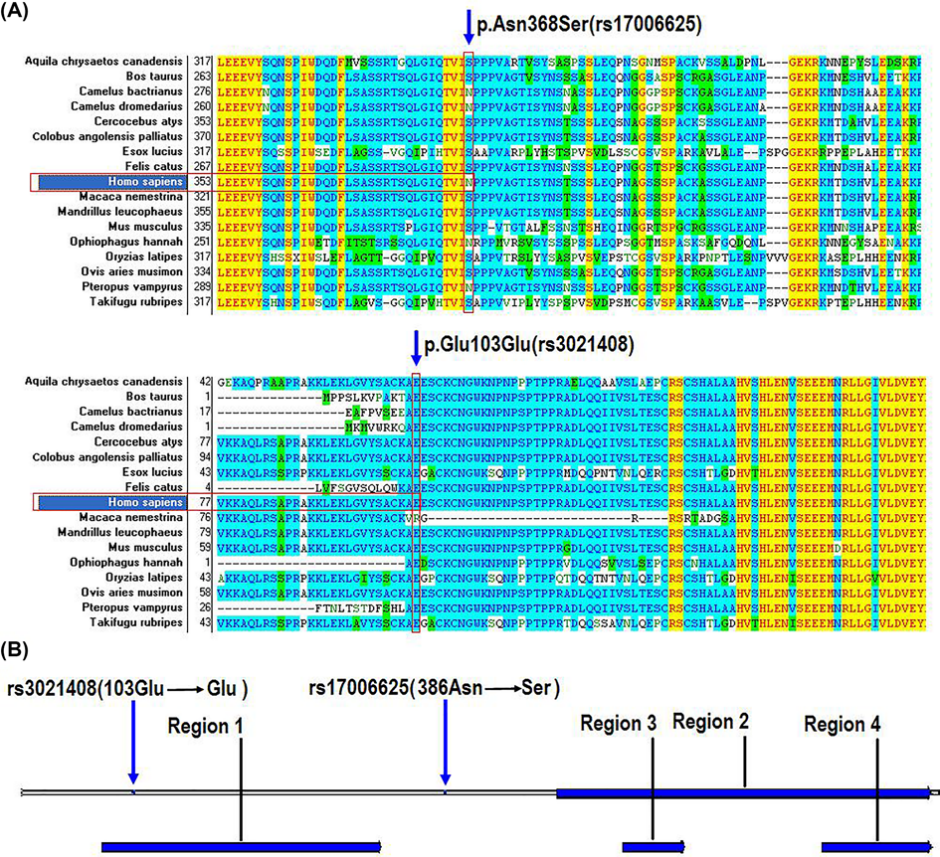
Multiple-sequence alignment and position analysis of rs3021408 and rs17006625 (**A**) Multiple-sequence alignment of KAT2B from birds, fishes and mammals (including *Homo sapiens, Pan troglodytes, Macaca mulatta* etc.). (**B**) Position analysis of the SNPs in the KAT2B protein.

We also compared the conservativeness of the KAT2B protein sequences from different species including birds, fishes, rodents and primates. Multiple-sequence alignment analysis showed that rs17006625 and rs3021408 were located within the conserved area of the KAT2B protein (Figure 2A), with rs17006625 being located between the first (Homology) and second (AT) functional domain of the KAT2B protein and rs3021408 within the first (Homology) functional domain (Figure 2B).

## Discussion

In the present study, we analyzed the transcribed regions and splicing sites of the epigenetic factor *KAT2B* gene in a large cohort of CHD patients and controls. We found that the variants rs3021408 and rs17006625 in the *KAT2B* gene were associated with the risk of CHDs in the Chinese Han population, suggesting the possible involvement of the *KAT2B* gene in the etiology of the disease.

The epigenetic and genetic factors affect many protein expression and metabolic changes in human embryonic stem cells and induced pluripotent stem cells. Those cells are perfect cell sources for human complex disease modeling and regenerative medicine [26]. The importance of epigenetic factors in human complex diseases can enable us to better understanding for those diseases, and may be used for diseases risk prediction and treatment [21].

During the formation of the human heart many genes and factors are strict temporal, spatial, and sequential expressed and translated [2]. The expression and translation of those genes or factors are regulated by those epigenetic factors [31]. The KAT2B family members are one of the epigenetic factors, have been found implicated in a variety of biological processes and human complex disease [31,32]. This work further emphasized the importance of epigenetic factors especially the KAT2B in the etiology of CHDs.

In the human or eukaryotic cells, the histone proteins are wrapped by DNA and formed the nucleosomes, but it is not only a packaging protein but also a regulatory protein [27]. When the histones are been chemical modified the chromatin structure can be altered, and the accessibility of DNA elements to transcription factors are increased or decreased, that can regulates the transcription of genes [33]. KAT2B is the CREBBP associated factor, that can acetylate many histones and non-histone proteins, and regulates many genes expression involved in cell proliferation [34,35]. It has been found that variations in the *KAT2B* gene promoter can reduce the risk of coronary heart disease mortality and restenosis [21], and the heterozygous for the low-risk allele patients had about 20 present lower risks of the cardiovascular events and homozygous allele individuals had about 40 present lower risks [21]. In this work, we found many creatures have the G/G allele (p.Ser368) of the rs17006625 and very few creatures have the A/A allele (p.Asn368) including human, Pteropus vampyrus, Ophiophagus Hannah, Camelus bactrianus and Camelus dromedarius. The interesting thing is that more congenital heart diseases patients were likely to have the A/A allele and for the normal population were more likely to have the A/G or G/G allele (Supplementary Table S1).

There are mainly three conserved functional domains in the KAT2B protein sequence, the homology domain, AT domain and bromodomain [36]. The homology domain is located in the N-terminal half of the protein, and the AT domain and bromodomain are located in the C-terminal half [37]. The central region of the AT domain mediates acetyl CoA binding and catalysis, and the N- and C-terminal regions of the AT domain mediates histone substrate specificity [38,39]. It has been found that the normal functions of the protein need the combined actions of homology and AT domains [40]. In this work, we found the rs17006625 (p.Ser368) was located between the homology and AT domains of the protein.

The KAT2B protein homology domain is also contained a potential E3 ligase activity region, that could functions as a ubiquitin E3 ligase for Hdm2, and promotes the p53 protein degradation [41]. So the homology domain of the KAT2B protein plays some roles in regulating cellular p53 levels, and KAT2B underlines the functional connections between cellular acetylation and ubiquitination machineries [42]. In this work, we found the location of the SNP rs3021408 was in the homology domain and E3 ligase activity region. All those showed the important roles of the homology and AT domains for the KAT2B protein, and this is may be the reasons why rs3021408 and rs17006625 in the *KAT2B* gene were associated with the risk of CHDs.

## Conclusion

We analyzed the transcribed regions and splicing sites of the epigenetic factors *KAT2B* gene between 400 Chinese Han CHD patients and 420 controls, and revealed a correlation between rs3021408 and rs17006625 in the *KAT2B* gene and risk of CHDs.

## Supplementary Material

Supplementary Figure S1 and Tables S1-S3

## Abbreviations

CDS: coding sequence
CHD: congenital heart disease
HAT: histone acetyltransferase
KAT2B: lysine acetyltransferase 2B
OD260/280: optical density 260/280
