# Supplementary material for: Identification of epigenetic factor KAT2B gene variants for possible roles in congenital heart diseases
Source: Biosci Rep. 2020 Apr 15;40(4):BSR20191779. doi: 10.1042/BSR20191779 (PMC7160239; doi:10.1042/BSR20191779)
Supplement: Supplementary Figure S1 and Tables S1-S3 [file BSR-2019-1779_supp.pdf]

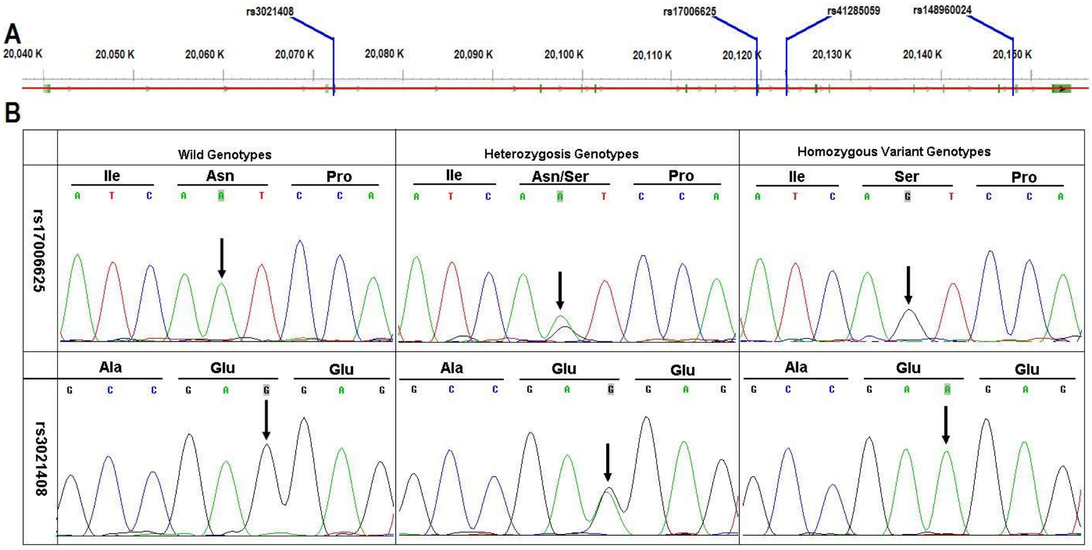

A
rs3021408
rs17006625
rs41285059
rs148960024
20,040 K
20,050 K
20,060 K
20,070 K
20,080 K
20,090 K
20,100 K
20,110 K
20,120 K
20,130 K
20,140 K
20,150 K
B
Wild Genotypes
Heterozygosis Genotypes
Homozygous Variant Genotypes
rs17006625
Ile
Asn
Pro
Asn/Ser
Ser
rs3021408
Ala
Glu
Glu

Figure_S1: Schematic diagrams and DNA sequence chromatograms. A: Schematic diagrams of rs3021408, rs17006625, rs148960024 and rs41285059 locations in the *KAT2B* gene; B: Three genotypes of DNA sequence chromatograms of 17006625 and rs3021408.

Table_S1: PCR primers used for *KAT2B* gene sequence analysis

Exon	***Forward primer***	***Reverse primer***	***Size***	***Tm***
1	GCCGCCACCACCATCAGC	CGGGCGACAAGAGCACTTCC	670	65.4
2	AGGCTAAGGCTCAAGAAC	CAAACTCTGAGGGCAAAA	477	48.3
3	GGGTTACCTGATAGTTGC	GGGAAGCCGTAGATAGAG	390	46.7
4	TGCTAATAACCACCAATC	CTAGGCAGCCCTATACTT	366	48.1
5	CAAGTGGTGATGTAAGGGTA	AAAGGGAACTGAGGGTCT	526	50.7
6	CCAGGGCTGCCCAACTAA	GCCGCTTTCACTCACTATCC	538	51.9
7	AGTCCCTGCTGTCTTTAC	ATCCTGGACATGCACAATCT	403	50.3
8	TGGGTTGGCAGTTATTGT	AAGTGGACCTGTGATGTG	532	50.9
9	TCTCAAAATGCTTTACCT	CTATGGCTGACTTTCACT	507	49.2
10	ATGGGAGTGCTGATGACA	CTAATCTGACCCGTGGAA	555	50.8
11	GGTCCTGGTTGGTAAAAT	AACGCTACCTGAGAACAT	298	47.1
12	ACAGTGCCTGGAACATAG	GATAACCCACAGCAAACC	349	48.9
13	CCCCTTTCTCCCATCTTA	CACAAACCACGGCATTAC	297	48.1
14	CTGAAAGGTTGGTGTTCC	AGAGTTTTACCCTATTGC	485	46.2
15-16	GAGACAGGCTGGAAACCG	CTTCCTACCCTAAGCAGA	630	49.2
17	GATTCCTTTCCCAGTCCC	AAACTAAACCACCTTGCT	340	48.0

Table _S2: PCR primers used for SNP statistical analysis

SNP	***Direction***	***primer***	***Size***	***Tm (℃)***
rs3021408	*Forward*	ATGGCTACAACTCCGACAG	412bp	51.2
	Reverse	ACCACTTCATGGACACCC		
rs17006625	*Forward*	GAGTTATAGTCCCTGCTGTC	299bp	51.0
	Reverse	CCTGGGTTTGCCTCAAGT		
rs148960024	*Forward*	GGGGCAGCGTTGTTAGTC	652bp	50.0
	Reverse	CTTTGATGGCTCTTGGGT		
rs41285059	*Forward*	GTTGGCGTGTATTATTTG	413bp	50.1
	Reverse	AAGTGGACCTGTGATGTG		

Table_S3: The genotype and allele frequency of SNP rs3021408 and rs17006625 in 400 CHD patients and 420 non-CHD controls

	Group		***Genotype frequency (%)***			***Allele frequency (%)***	
rs3021408	Genotype		C/C	C/T	T/T	C	T
	CHD	400	102(25.5)	192(48.0)	106(26.5)	396(49.5)	404(50.5)
	Controls	420	140(33.3)	206(49.0)	74(17.6)	486(57.9)	354(42.1)
rs17006625	Genotype		A/A	A/G	G/G	A	G
	CHD	400	316(79.0)	80(20.0)	4(1.0)	712(89.0)	88(11.0)
	Controls	420	302(71.9)	111(26.4)	7(1.7)	715(85.1)	125(14.9)
